# Flavonoid *C*-glucosides Derived from Flax Straw Extracts Reduce Human Breast Cancer Cell Growth *In vitro* and Induce Apoptosis

**DOI:** 10.3389/fphar.2016.00282

**Published:** 2016-08-31

**Authors:** Magdalena Czemplik, Justyna Mierziak, Jan Szopa, Anna Kulma

**Affiliations:** ^1^Department of Physico-Chemistry of Microorganisms, Institute of Genetics and Microbiology, University of Wrocław, WrocławPoland; ^2^Faculty of Biotechnology, University of Wrocław, WrocławPoland; ^3^Linum Foundation, WrocławPoland; ^4^Department of Genetics, Plant Breeding and Seed Production, Wrocław University of Environmental and Life Sciences, WrocławPoland

**Keywords:** flax straw, flavonoid *C*-glucosides, MCF-7, human breast carcinoma cells, apoptosis

## Abstract

Flax straw of flax varieties that are grown for oil production is a by product which represents a considerable biomass source. Therefore, its potential application for human use is of high interest. Our research has revealed that flax straw is rich in flavonoid *C*-glucosides, including vitexin, orientin, and isoorientin. The objective of this study was to evaluate the cytotoxicity and possible proapoptotic effect of flax straw derived *C*-glucosides of flavonoids in the human breast adenocarcinoma cell line (MCF-7). The effects of flax straw derived flavonoid *C*-glucosides on cell proliferation of MCF-7 cells were evaluated by 3-[4,5-dimethylthiazol-2-yl]-2,5-diphenyl-tetrazolium bromide (MTT) and sulforhodamine B assays. The expression of apoptosis-related genes was assessed by real-time PCR. Our data revealed that flax *C*-glucosides as well as pure compounds are cytotoxic toward MCF-7 cells and inhibit their proliferation. Moreover, the induction of apoptosis was correlated with the changes in the mRNA level of pro-apoptotic genes. Increased expression of bax and caspase-7, -8, and -9 and decreased mRNA expression of bcl-2 was observed, whereas the mRNA levels of p53 and mdm2 were not altered. These results clearly demonstrated that flax straw metabolites effectively induced growth inhibition and apoptosis in human breast adenocarcinoma cells.

## Introduction

Flavonoids are among the most abundant polyphenols in plants. Based on their structures, they can be grouped into several subclasses such as flavonols, flavones, isoflavones, flavanones, and flavan-3-ols. Normally they are accumulated in the vacuoles of plant tissues as *O*-linked glycosidic conjugates, but also as *C*-glycosides (Harborne, 1993). The flavonoid glycosides mainly occur as 3 or 7 *O*-glycosides, but the C-5, 6, 8 and 4 positions are glycosylated as well. The transformation of flavonoid aglycones to glycosides plays a crucial role in flavonoid biosynthesis, and their glycosylation increases their solubility and stability relative to flavonoid aglycones (Plaza et al., 2014). The sugars of *C*-glycosides are attached directly to the flavonoid skeleton by a C–C bond that is resistant to acid hydrolysis. Most flavonoid *C*-glycosides are flavones that have been found in bryophytes, ferns, gymnosperms, and angiosperms (Harborne, 1993). In cereals such as rice (*Oryza sativa*), wheat (*Triticum aestivum*), and maize (*Zea mays*), *C*-glycosylflavones are the main class of accumulated flavonoids (Brazier-Hicks et al., 2009). It was found that in buckwheat the direct precursors of flavone-*C*-glycosides are 2-hydroxyfl-avanones, which underwent catalyzed *C*-glucosylation (Kerscher and Franz, 1988). Thus, the flavanones, which are core intermediates of the flavonoid pathway, are the most likely precursors of *C*-glucosides. It is known that cereal crops synthesize *C*-glucosylated flavones through the activity of *C*-glycosyltransferases and dehydratase acting on activated 2-hydroxyflavanones (Brazier-Hicks et al., 2009).

In plants, flavone-*C*-glycosides function as antioxidants (Kitta et al., 1992), insect feeding attractants (Kim et al., 1985), antimicrobial agents (Dinda et al., 2006), and promoters of mycorrhizal symbioses (Akiyama et al., 2002). It was observed that accumulation of *C*-glycosylflavones was enhanced by UV-B light in a UV-tolerant rice cultivar but absent in a susceptible cultivar, which might suggest their possible UV light protective roles (Markham et al., 1998). Interestingly, they also possess some favorable activities for humans, as they can protect cells from oxidative stress and counteract inflammation (Manthey et al., 2001; Kim et al., 2005). For example, isovitexin extracted from rice bran was shown to prevent reactive oxygen species damage in an *in vitro* system (Lin et al., 2002) and was able to reduce the production of hydrogen peroxide induced by LPS in mouse macrophages (Lin et al., 2005). The flavone *C*-glycosides from blueberry are able to reduce IL-8 production, which indicates their potential as anti-inflammatory agents (Flores et al., 2012). There are some reports on the important role of flavonoid *C*-glycosides in cancer development. Dietary flavone was shown to alter expression of genes that regulate cancer cell proliferation, cycle, and apoptosis (Ullmannova and Popescu, 2007). Tricin has *in vitro* antiproliferative activities toward breast and colon cancer cell lines at submicromolar concentrations (Hudson et al., 2000). Vitexin is suggested to serve as a therapeutic agent for the treatment of human leukemia, as it promoted the expression of cleaved caspase-3 and cleaved caspase-9 in leukemia cells and simultaneously reduced Bcl-2 expression and therefore induced apoptosis (Lee et al., 2012).

Various flavonoid *C*-glucosides and di-*C*-glucosides were found in flax (Dubois and Mabry, 1971), including orientin and isoorientin and their derivatives (Wagner et al., 1972; Mierziak et al., 2014). Flax straw was found to be a rich source of valuable metabolites, including *C*-glycosylated flavonoids. Flax straw is one of the by-products of flax oil production and increases the biomass reservoir; hence, its putative application for human use is of high interest.

In this study, we identified and determined the level of *C*-glycosylated flavonoids in flax straw extracts. For the purpose of our research we used different genetically modified flax types in which the secondary metabolite accumulation in the plant organs was increased. Three genetically modified flax types (named W94, GT4, and L9) were generated and described previously (Lorenc-Kukuła et al., 2005, 2009; Boba et al., 2011). W92 flax overexpresses three genes of the phenylpropanoid pathway encoding chalcone synthase (CHS), chalcone isomerase (CHI), and dihydroflavonol reductase (DFR). This modification resulted in an increase in phenylpropanoid content: flavonoids (kaempferol and quercetin), anthocyanins, phenolic acids (coumaric, ferulic, and synaptic acids) and lignan (Żuk et al., 2011). GT4 flax overexpresses the glucosyltransferase gene and is characterized by overproduction of phenylpropanoids such as kaempferol and quercetin glycosides, anthocyanins, proanthocyanidins, phenolic acids and their glucoside derivatives, and SDG (Lorenc-Kukuła et al., 2009; Czemplik et al., 2012). L9 flax, also used in this study, was obtained by suppression of the endogenous flax gene coding for lycopene β-cyclase, which resulted in a decrease in carotene content, but also in an increase in accumulation of other terpenoids and tocopherols (Boba et al., 2011).

Combinations of natural molecules or extracts that effectively inhibit tumor development and progression are nowadays actively researched (Pan et al., 2010). In this study, we investigated the *in vitro* effect of straw extracts from three transgenic flax types (W92, GT, and L) and one non-transgenic one (Linola) on the growth, proliferation and apoptosis of cells from the human breast cancer cell line MCF-7. We also tested the effects of the pure compounds of the main constituents of flax straw on human breast cancer cells.

## Materials and Methods

### Plant Material

Flax seeds (cv. Linola 947) were obtained from the Flax and Hemp Collection of the Institute of Natural Fibers in Poland. The GT plants overexpress the glucosyl transferase gene SsGT1 from *Solanum sogarandinum* under a seed-specific napin promoter. W92 plants overexpress three genes of the phenylpropanoid pathway: CHS, CHI, and DFR. L plants were transformed with lycopene b-cyclase (lcb) from *Arabidopsis thaliana*. GT transgenic plants line #4 (GT4) and line #5 (GT5) and W92 transgenic plants line #40 (W92.40) and line #72 (W92.72), L transgenic plants line #9 (L9) and Linola, the non-transgenic control type, were grown in a field and were harvested 4.5 months after sowing. After the separation of seed capsules, the straw was used for further experiments.

### Phenolic Component Extraction and UPLC Analysis

A 5 g amount of flax straw was ground using a Retsch mill and extracted three times with methanol. Fractions were pooled and evaporated at 40°C under a vacuum and then resuspended in 1 mL of water. The extract was analyzed on a Waters Acquity UPLC system with a 2996 PDA detector, using an Acquity UPLC column BEH C18, 2.1100 mm, 1.7 μm. The mobile phase was acetonitrile (solution A) and 0.1% formic acid (solution B) as the eluent. The separation was done at 4 ml/min flow ratio with gradient flow: 1 min—95% A and 5% B, 2–12 min—gradient to 70% A and 30% B, 12–15 min—gradient to 0% A and 100% B, and 15–17 min—gradient to 95% A and 5% B. The compounds were identified based on the retention times and absorbance spectra, and their quantities were calculated in comparison to the standards (Sigma-Aldrich, USA). The detection was done at *k* = 280 nm.

### Cell Culture

The human breast adenocarcinoma cell line (MCF-7) was obtained from the Laboratory of Nuclear Proteins of the Faculty of Biotechnology, University of Wrocław, Poland and was grown as a monolayer in Eagle Minimum Essential Medium (Lonza, Switzerland) supplemented with 10% fetal bovine serum (Lonza, Switzerland), 1% L-glutamine (Invitrogen, USA) and a 1% antibiotic mixture (Invitrogen, USA) at 37°C in a 5% CO_2_ atmosphere.

### Cell Proliferation Assay

Cells were seeded in a 96-well plate at a concentration of 5 × 10^3^ cells/mL. After 24 h, GT4, GT5, W92.40, W92.72, L and Linola flax straw extracts were added to the plate at varying volumes: 5, 10, and 20 μL. Each flax type straw extract was prepared from an equal amount (5 g) of dry weight of straw and the flavonoid content in each sample was presented in **Table 2**. Additionally, the pure compound standards of vitexin (0.2, 0.4, 0.6, 0.8, 1, 1.2, 1.4 μg), isoorientin (20, 40, 60, 80, 100, 120, 140, 160 μg), and orientin (0.1, 0.25, 0.5, 1, 1.5 μg) were added at different concentrations corresponding to the amount in the flax straw extracts. To assess the proliferation potential, MCF-7 cells were incubated and assayed after 24 and 48 h. After this period of treatment, 10 μL of MTT stock solution (4 mg/mL) was added to each well to give a total reaction volume of 550 μL. After 4 h of incubation, the medium with MTT solution was removed from the plate. The formazan crystals in each well were dissolved in 50 μL of DMSO and incubated for 30 min with gentle shaking. The absorbance at 540 nm was read on a Varioskan Flash Microplate Reader (Thermo Scientific, USA). The MTT assay was performed in four repetitions. The results were presented as a % in referrence to the control (100%).

### Cell Cytotoxicity Assay

*In vitro* cytotoxicity against human cancer cell line MCF-7 was determined using the sulforhodamine B assay (SRB) as described previously (Skehan et al., 1990). Cells were seeded in a 96-well plate at concentration of 5 × 10^3^ cells/mL. After 24 h, GT4, GT5, W92.40, W92.72, L and Linola flax straw extracts were added to the plate at varying volumes: 5, 10, and 20 μL. Each flax type straw extract was prepared from an equal amount (5 g) of dry weight of straw and the flavonoid content in each sample was presented in **Table 2**. Additionally, the pure compound standards of vitexin (0.2, 0.4, 0.6, 0.8, 1, 1.2, 1.4 μg), isoorientin (20, 40, 60, 80, 100, 120, 140, 160 μg), and orientin (0.1, 0.25, 0.5, 1, 1.5 μg) were added at different concentrations corresponding to the amount in the flax straw extracts. To assess the cytotoxicity, the MCF-7 cells were incubated and assayed after 24 and 48 h. After incubation, the cells were fixed by gently layering trichloroacetic acid (50 μL/well, 50% w/v) on top of the medium in all the wells and incubated at 4°C for 1 h. The plates were washed five times with distilled water and air-dried. The staining was performed with sulforhodamine B dye (0.4% w/v in 1% acetic acid, 50 μL/well). The unbound dye was washed five times with 1% acetic acid and the plates were air-dried. The adsorbed dye was dissolved in Tris buffer (150 μL/well, 10 mM) and the plates were gently shaken for 10 min on a mechanical shaker. The absorbance at 530 nm was read on a Varioskan Flash Microplate Reader (Thermo Scientific, USA). The cytotoxicity effect was calculated by subtracting the mean OD values of the respective blank from the mean OD value of the experimental set. The percentage growth in the presence of the test extract was calculated considering the growth in the absence of any test extracts as 100%.

### RNA Purification and Real-Time PCR Analysis

The expression of apoptosis-related genes, such as bcl-2, bax, caspase-7, -8 and -9, p53 and mdm2, was determined by real- time PCR (RT-PCR). MCF-7 cells were seeded on 24- well plate at concentration of 20 × 10^5^ cells/mL. Linola flax straw extracts (Lin.1, Lin.2, and Lin.3) were added to the plate. After 24 h, the cells were washed twice with PBS and then, the total RNA isolation was performed using the RNeasy Plus Kit (QIAGEN) following the manufacturer’s protocol. The remaining DNA was removed via DNase I (Invitrogen) treatment. Then, RNA was used as a template for cDNA synthesis using a High Capacity cDNA Reverse Transcription Kit (Applied Biosystems). Real-time PCR reactions were carried out using a DyNAmo SYBR Green qPCR Kit (Thermo Scientific) on an Applied Biosystems StepOnePlus Real-Time PCR System. Reaction conditions were designed according to the kit manufacturer’s instructions. The oligonucleotide primer pairs for RT-PCR used in the present study were: bcl2, 5′-TGGCCTTCTTTGAGTTCG-3′ (sense), 5′-GTACAGTTCCACAAAGGCAT-3′ (antisense); bax, 5′-CGAACTGGACAGTAACATGG-3′ (sense), 5′-CAGTTTGCTGGCAAAGTAGA-3′ (antisense); caspase-7, 5′-CATGCGATCCATCAAGACCA-3′ (sense), 5′-GGAAGCACTTGAAGAGCG-3′ (anti-sense); caspase-8, 5′-AATTAATAGACTGGATTTGCTGATTAC-3′ (sense), 5′-CCTCAATTCTGATCTGCTCAC-3′ (anti-sense); caspase-9, 5′-TTGTCGAAGCCAACCCTA-3′ (sense), 5′-GCCAAATCTGCATTTCCC-3′ (antisense); p53, 5′-ACATGACGGAGGTTGTGA-3′ (sense), 5′-CACCACCACACTATGTCG-3′ (antisense); mdm2, 5′-AAGGAGAGCAATTAGTGAGAC-3′ (sense), 5′-TGCTACTGCTTCTTTCACAAC-3′ (anti-sense), annexin V, 5′-CTTTATTTCAGGCTGGAGAACTTA-3′ (sense), 5′-AATCCTGATATAGTCATGTACTTGT-3′ (antis-ense). Reactions were carried out in three replicates. The GAPDH gene was used as a reference gene with the following primers: 5′-AGGTCGGAGTCAACGGAT-3′ (sense) 5′-TCCGGAAGATGGTGATG-3′ (antisense). All used primers are listed in Supplementary Table S1. The changes in transcript levels were presented as the relative quantification to the reference gene.

### Detection of Apoptosis by Annexin V Labeling

The detection of surface phosphatidylserine by FITC-annexin V binding was performed with use of Annexin V-FITC Fluorescence Microscopy Kit (BD Pharmingen^TM^). MCF-7 cells were seeded on 24-well plate at concentration of 20 × 10^5^ cells/mL. After 24 h, Linola flax straw extracts (Lin.1, Lin.2, and Lin.3) were added to the plate. The positive control consisted cells treated with 1 μM of staurosporine (known to cause an apoptotic effect on a variety of human tumor cell lines) and the negative control consisted non-treated cells. After 24 h of treatment, the -treated, positive- and negative- control cells were washed twice with cold phosphate-buffered saline (PBS) and once with annexin binding (AB) buffer. Cells were then incubated for 15 min with phosphatidylserine labeling solution (Annexin V-FITC diluted 1:10 in AB buffer). Additionally, the nuclear morphology was analyzed by staining the cells with 5 μg/ml Hoechst (Invitrogen, Matrigel^TM^) in PBS for 15 min. Cells were then washed with AB buffer and observed immediately by fluorescence microscopy. An Olympus FV500 confocal laser scanning microscope was used for fluorescence observation.

### Western Blotting Analysis

MCF-7 cells were seeded on 24- well plate at concentration of 20 × 10^5^ cells/mL. Linola flax straw extract (Lin.3) was added to the plate. After 24 h of incubation the cells were washed twice with PBS and then scraped at 4°C in lysis buffer: 100 mM Tris-HCl, pH 7.4, 1% Triton X-100 and protease inhibitor cocktail (Bioshop Canada, Inc.). Samples were sonicated three times for 30 s and stored at -20°C for further experiments. The protein concentration of the samples was determined by the standard Bradford (Bradford, 1976) procedure. Identical amounts of protein (50 μg) were separated on 12% sodium dodecyl sulfate-polyacrylamide gel electrophoresis (SDS-PAGE) and blotted electrophoretically onto nitrocellulose membranes (Schleicher and Schuell). After the transfer, the membrane was sequentially incubated with a blocking buffer (5% dry milk) and then with monoclonal mouse anti-caspase 7 antibody (1: 1000 dilution) and polyclonal rabbit anti-caspase 8 antibody (1: 1000 dilution) overnight at 4°C. An antibody for GAPDH (Sigma-Aldrich, USA) was used as an internal control. Alkaline phosphatase-conjugated goat anti mouse IgG and anti-rabbit IgG served as a second antibody, and was used at a dilution of 1: 25000. Immunoreactivity was visualized by Alkaline Phosphatase Substrate Kit (Bio-Rad) according to the protocol supplied with the kit.

### Statistical Analysis

Each test procedure (MTT and SRB test, RT-PCR) was performed three times, whereas in each single test, four repetitions were performed. The results were averaged and a standard deviation was calculated so that each bar seen in the diagrams represents the mean value of four separate experiments ± standard deviation. Analysis of variance (ANOVA) was performed using the Tukey test. Statistica 9 (Statsoft) software was used for the statistical analysis.

## Results

### Identification and Quantification of Flavonoid *C*-glucosides in Flax Straw

The UPLC analysis of the methanol extracts of flax straw revealed the presence of three flavonoid *C*-glucosides: vitexin, orientin, and isoorientin. The amounts of those components were determined in straw from transgenic flax type GT4, GT5, W92.40, W92.72, L9 and a control, non-transgenic flax line (Linola). The results are presented in **Table 1**. Generally, the level of the three identified flavonoid *C*-glucosides in all flax types did not differ dramatically, but some differences were observed. In all flax types the most abundant compound was isoorientin, which reached 9 mg/gFW in Linola type. In all transgenic types, the level of isoorientin was slightly lower, with the lowest amount in the W92.72 type (4.53 mg/gFW). Vitexin was the most elevated in the GT4 flax straw (0.083 mg/gFW), and was lower in the W92.72 type (0.037 mg/gFW) in comparison to the control. W92.72 flax straw was characterized by the highest level of orientin (0.062 mg/gFW). For the cell *in vitro* studies, we used three different volumes (20, 10, and 5 μl) of straw extracts derived from the same amount of tissue (5 g) of each flax type, and their biochemical composition is presented in **Table 2**.

**Table 1:** Flavonoid *C*-glucosides composition in the flax straw extracts.

Flax type	Vitexin (mg/gFW)	Orientin (mg/gFW)	Isoorientin (mg/gFW)
Linola	0.066 ± 0.0054	0.052 ± 0.004	9.01 ± 0.46
GT4	0.083 ± 0.0092	0.057 ± 0.0011	8.01 ± 0.71
GT5	0.057 ± 0.0012	0.030 ± 0.0078	5.87 ± 0.63
W92.40	0.049 ± 0.005	0.062 ± 0.0022	7.23 ± 0.26
W92.72	0.037 ± 0.0043	0.045 ± 0.0061	4.53 ± 0.45
L9	0.054 ± 0.0018	0.053 ± 0.0095	7.06 ± 0.13

**Table 2:** Biochemical composition of flax straw extracts used for cell *in vitro* studies.

	Compound (μg)	20 μl	10 μl	5 μl
Linola	Vitexin	1.32	0.66	0.33
	Orientin	1.04	0.52	0.26
	Isoorientin	180.36	90.18	45.09
GT4	Vitexin	1.66	0.83	0.41
	Orientin	1.01	0.50	0.25
	Isoorientin	160.22	80.11	40.05
GT5	Vitexin	1.14	0.57	0.28
	Orientin	0.61	0.30	0.15
	Isoorientin	117.53	58.76	29.38
W92.40	Vitexin	0.98	0.49	0.24
	Orientin	1.24	0.62	0.31
	Isoorientin	144.63	72.31	36.15
W92.72	Vitexin	0.75	0.37	0.18
	Orientin	0.90	0.45	0.22
	Isoorientin	90.74	45.37	22.68
L9	Vitexin	1.09	0.54	0.27
	Orientin	1.07	0.53	0.26
	Isoorientin	141.37	70.68	35.34

### Effects of Flax Straw Extracts on Proliferation of Human Breast Carcinoma Cells and Cytotoxic Effect

**FIGURE 1:**
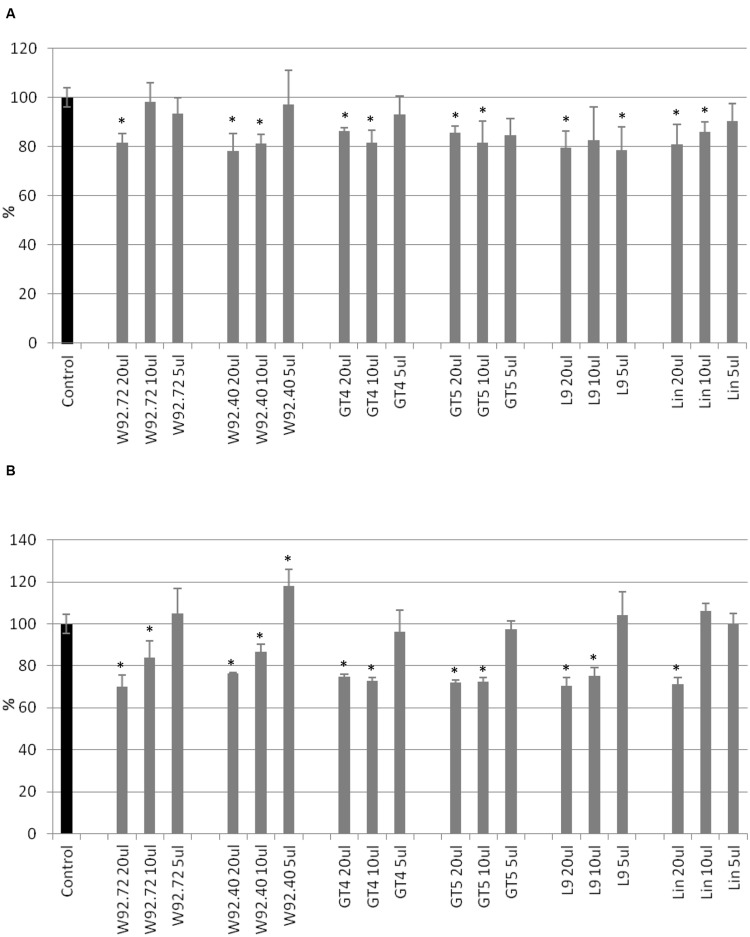
**The effect of flax straw extracts on MCF-7 cells in *in vitro* MTT test.** 20, 10, and 5 μl of straw extracts from W92.72, W92.40, GT4, GT5, L9 and Linola flax types were added to the MCF-7 cell culture. The MTT assay was performed in four repetitions for each sample after 24 h **(A)** and 48 h **(B)** of incubation.

The proliferation of human breast carcinoma cells treated with flax straw extracts was assessed using the MTT test (**Figure 1**). The proliferation level of MCF-7 cells was diminished to 80% in comparison to the control, after 24 h of treatment, for most of the flax types tested. Generally, no significant differences among different flax types were observed (**Figure 1A**). After 48 h of treatment, the inhibitory effect was stronger, and 10 and 20 μl volumes of all preparations tested lowered the proliferation level by around 30%. The most effective were 20 μl of W92.72 and L9 extracts (**Figure 1B**). However, the lower volumes of flax straw extracts exhibited a pro- proliferative effect on MCF-7 cells after 48 h of treatment. A 5 μl of W92.72, W92.40, GT4, GT5, L9, 10 and 5 μl of Linola straw extract induced proliferation potential with the highest increase of 18% for 5 μl of W92.40 straw extract (**Figure 1B**).

**FIGURE 2:**
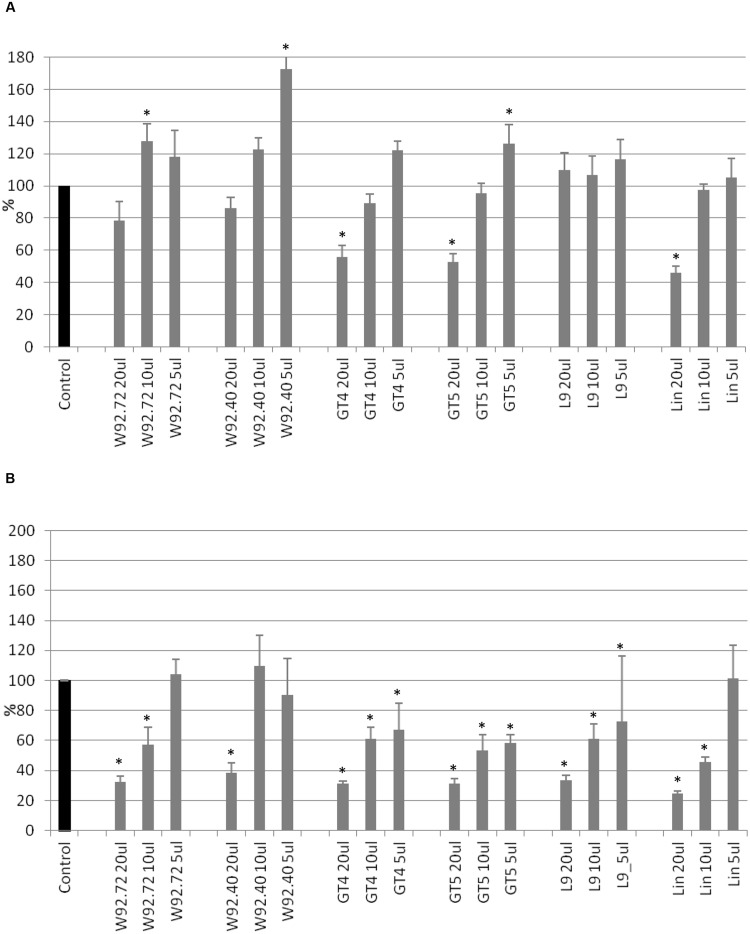
**The effect of flax straw extracts on MCF-7 cells in *in vitro* SRB test.** 20, 10, and 5 μl of straw extracts from W92.72, W92.40, GT4, GT5, L9 and Linola flax types were added to the MCF-7 cell culture. The SRB assay was performed in four repetitions for each sample after 24 h **(A)** and 48 h **(B)** of incubation.

Cell cytotoxicity was assessed using the SRB assay. After 24 h of treatment, addition of 20 μl of flax straw extracts (apart from the L9 type) resulted in a cytotoxic effect of up to 46% for Linola extract, in comparison to the control (**Figure 2A**). For smaller extract volumes such an effect was not observed, but on the contrary, the anti- cytotoxic effect was noticed for the following samples: 10 and 5 μl of W92.72, W92.40, GT4, GT5, Linola and all samples of L9 flax straw extracts. After 48 h of treatment, the 5 μl of W92.72, 10 and 5 μl of W92.40 and 5 μl of Linola straw extract exhibited no cytotoxic effect on human breast cancer cells (**Figure 2B**). The strongest cytotoxic effect was observed after 48 h of treatment for all other preparations. Treatment of MCF-7 cells with the highest amount of straw extracts resulted in a 70% decrease in protein content in comparison to the non-treated cells. The Linola preparation was again the most effective (**Figure 2B**). The results showed that each of the tested flax straw extracts exhibited anti-cancer effects on MCF-7 cells.

### Influence of Main Constituents of Flax Straw Extracts on Human Breast Cancer Cells

**FIGURE 3:**
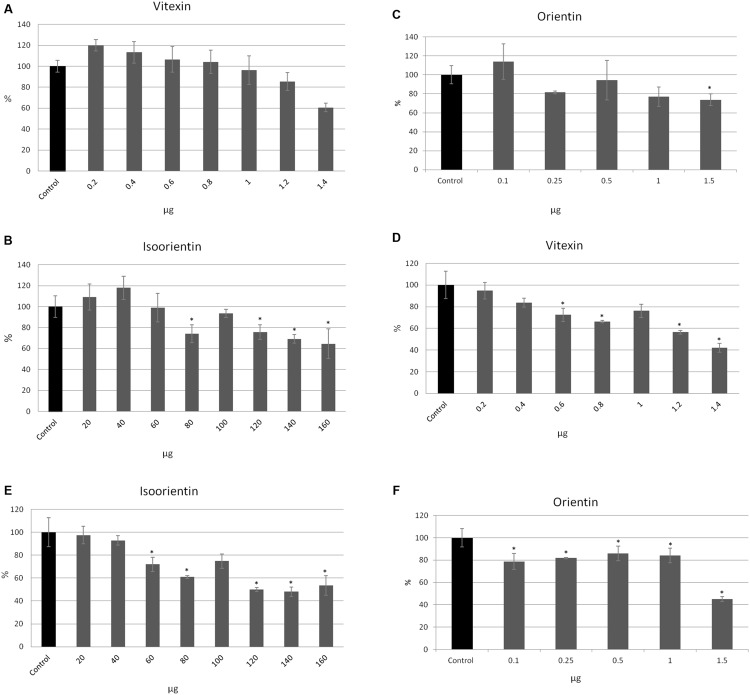
**The effect of pure compounds of flavonoid *C*-glucosides on proliferation of MCF-7 cells.** Vitexin, isovitexin and orientin were added to the MCF-7 cell culture in different ranges of amounts (μg). The MTT assay was performed in four repetitions for each sample after 24 h **(A–C)** and 48 h **(D–F)** of incubation.

To investigate which of the flax straw extract components might be lower proliferation of MCF-7 cells, we verified the activity of the three main C-flavonoids: orientin (O), isoorientin (I), and vitexin (V). The amounts of the tested compounds correspond to the amounts in the previously tested preparations. The proliferation of MCF-7 cells in the MTT test was significantly decreased in the higher amounts of tested compounds, and this effect was stronger after 48 h of incubation. The vitexin, isoorientin, and orientin reduced MCF-7 proliferation by up to 40% in comparison to the control after 24 h of treatment (**Figures 3A–C**). The inhibition was stronger after 48 h of treatment and showed that vitexin reduced proliferation by up to 40% of the control value, while isoorientin inhibited proliferation by up to 50% (**Figures 3D,E**), and orientin by up to 55% (**Figure 3F**). However, the lower amounts of the tested compounds exhibited a stimulatory effect on the proliferation potential of MCF-7 cells. Vitexin (0.2, 0.4, 0.6 μg) caused the slight increase in the proliferation potential after 24 h of treatment, as well as isoorientin (20, 40 μg) and orientin (0.1 μg; **Figures 3A–C**). Such an effect was not observed after 48 h of treatment (**Figures 3D–F**).

**FIGURE 4:**
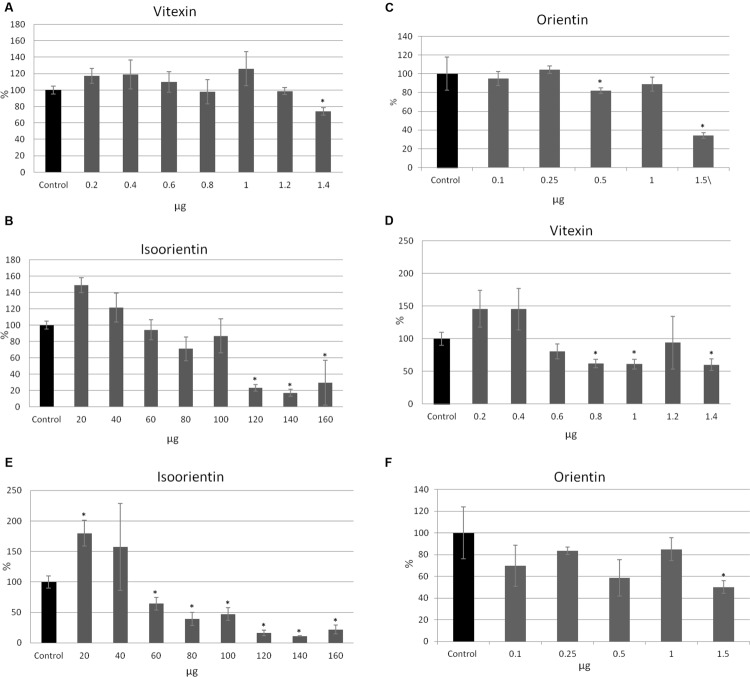
**The effect of pure compounds of flavonoid *C*-glucosides on MCF-7 cells in *in vitro* SRB test.** Vitexin, isovitexin and orientin were added to the MCF-7 cell culture in different ranges of amounts (μg). The SRB assay was performed in four repetitions for each sample after 24 h **(A–C)** and 48 h **(D–F)** of incubation.

All tested compounds exhibited a cytotoxic effect on MCF-7 cells. Interestingly, lower amounts of vitexin (0.2, 0.4, 0.6, 1 μg after 24 h, and 0.2, 0.4 μg after 48 h) and isoorientin (20, 40 μg after 24 and 48 h) caused an anti-cytotoxic effect (**Figures 4A,B,D,E**). For 0.8 and 1.2 μg of vitexin in the 24 h of treatment no significant changes were observed (**Figure 4A**). Similarly, for treatment with 0.1, 0.25, and 1 μg of orientin no significant alteration occured. After 24 h of treatment, addition of 1.4 μg of vitexin, 120, 140, and 160 μg of isoorientin and 0.5 and 1.5 μg of orientin resulted in a cytotoxic effect up to 27, 79, 83, and 66%, respectively, in comparison to the control (**Figures 4A–C**). The strongest effect was observed after 48 h of treatment (**Figures 4D–F**). After 48 h of treatment, addition of 0.6–1.4 μg of vitexin, 60–160 μg of isoorientin, and 0.1, 0.5, 1.5 μg of orientin resulted in a cytotoxic effect. The strongest effect was noted for 140 μg of isoorientin and was up to 86%, in comparison to the control (**Figures 4E**). These results indicate that all of the tested *C*-glucosyl derivatives of flavonoids exhibited a significant inhibitory effect toward MCF-7 cells.

### Flax Straw Extract Promoted Expression of Pro-apoptotic Genes of Human Breast Cancer Cells

To explore whether flax straw extracts induced expression of pro-apoptotic genes, the quantitative analysis of expression of apoptosis-related genes in MCF-7 cells was performed. Real-time PCR analyses were used to measure the mRNA expression of Bcl-2, Bax, caspase-7, -8, -9, p53, and mdm2, as well as the expression of the GAPDH (an internal standard), after the treatment of human breast cancer cells with the flax straw extract. As the previous experiments did not show significant differences among individual flax extracts regarding their influence on MCF-7 cells, for the purpose of this study, the Linola flax straw extract was chosen. Its effect on apoptosis of human carcinoma breast cancer cells was studied after 6, 12, and 24 h of treatment. For the experiment three different volumes of Linola flax straw extracts were used (Lin.1, Lin.2, Lin.3), which corresponded to the amounts in the previous experiments of this study.

**FIGURE 5:**
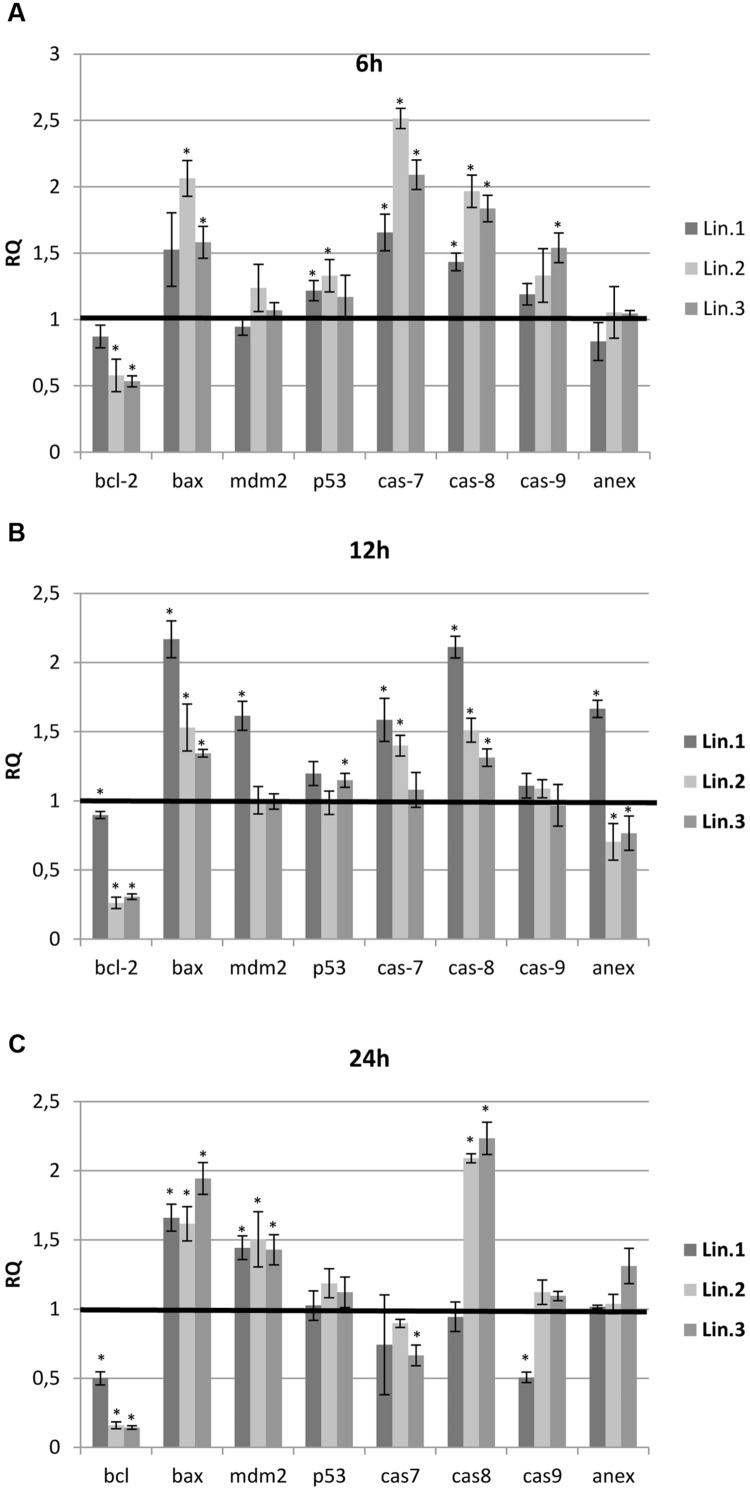
**Expression levels of genes involved in the apoptosis of MCF-7 cells.** The mRNA level of bcl-2 (B-cell CLL/lymphoma 2), bax (BCL2-associated X protein), p53, mdm-2 (MDM2 proto-oncogene), cas 7 (caspase 7), cas 8 (caspase 8), cas 9 (caspase 9) and annexin V(anex) in MCF-7 cells treated with Linola flax straw extracts (Lin.1, Lin.2, Lin.3) at 6 h **(A)**, 12 h **(B)**, and 24 h **(C)** was obtained from the real-time RT-PCR analysis. GAPDH was used as a reference gene and the transcript levels were normalized to those of the control non-treated cells (*C* = 1; not presented in the figure). Data represent the mean ± standard deviations from three independent experiments.

In all samples tested, the treatment with flax straw extracts resulted in significant changes in the expression of the bcl-2 and bax genes. A reduction in the transcript level of the bcl-2 gene was observed in all samples, with the lowest level at 24 h for Lin.2 and Lin.3 samples (**Figure 5C**). In contrast, the bax gene was overexpressed in all samples tested up to twofold. To determine the caspases involved in apoptosis enhancement, the analysis of expression level was performed for caspase-7, -8, and -9. At 6 h, the mRNA level of all caspases was elevated in all samples (**Figure 5A**). At 12 h, the caspase-7 and -8 were characterized by an increase of mRNA level, while caspase-9 exhibited no significant changes. At 24 h, the expression level of caspase-8 was elevated, whereas caspase-7 and -9 expression levels were not altered or slightly lowered (**Figure 5C**). Some differences were also observed in the expressions of p53 and mdm2 genes of flax straw treated MCF-7 cells. A slight increase was observed in mRNA level for the p53 gene, while the mdm2 gene was increased in all samples at 24 h (**Figure 5C**). The level of annexin V mRNA was not significantly altered upon flax straw extracts treatment. However, the increase in the expression level of this gene was observed for Lin1 extract after 12 h of treatment (**Figure 5B**).

### Induction of Apoptosis by Flax Straw Extract

**FIGURE 6:**
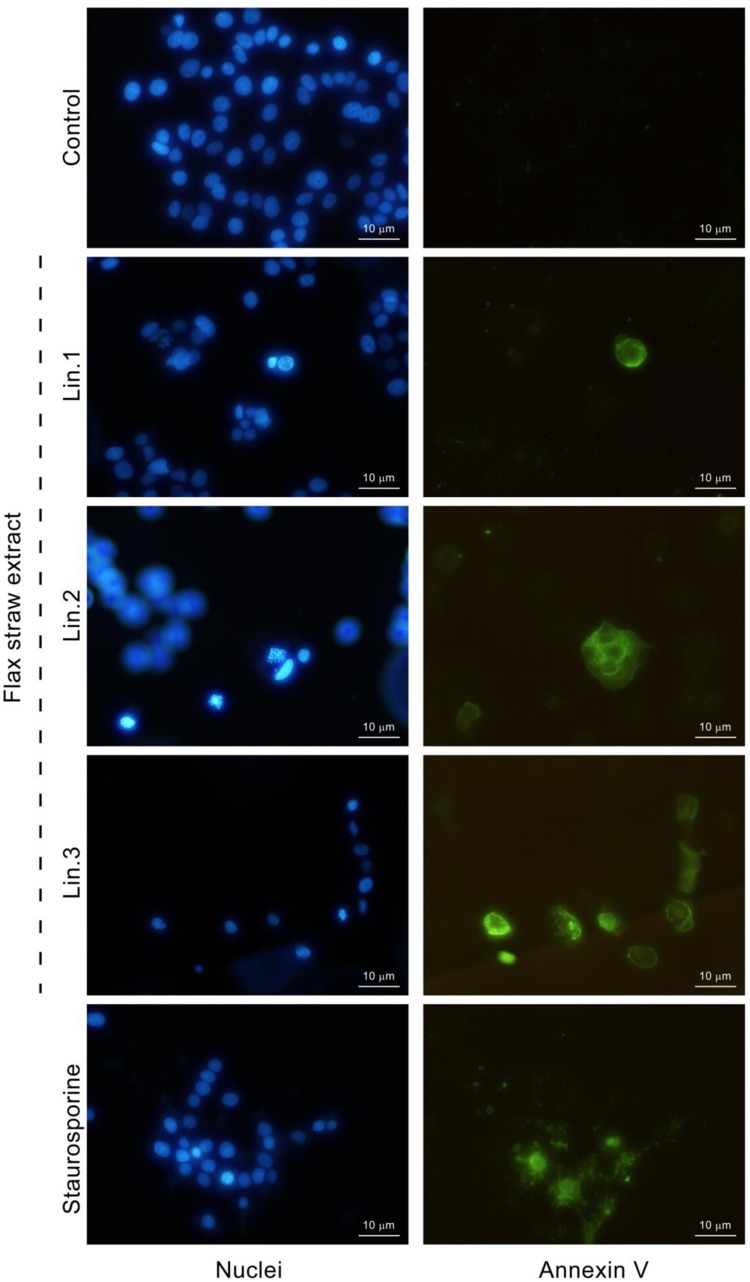
**Induction of apoptosis by flax straw extract in MCF-7 cells.** Detection of externalized phosphatidylserine by annexin V-fluorescein (right column). Nuclei were stained with Hoechst 33342 (left column). Results were visualized by fluorescence microscope and compared with those observed in the presence of the known apoptosis inducer staurosporine (1 μM).

To determine if the flax straw extract has an apoptotic effect, the annexin V assay was used to detect phosphatidylserine in the outer leaflet of the plasma membrane. Phosphatidylserine is localized in the inner leaflet of the plasma membrane, but while the cells undergo apoptosis, the asymmetry of the cell membrane is lost and phosphatidylserine is then also found on the outer surface of the cell membrane. Annexin V is a protein that binds to phosphatidylserine in the presence of calcium ions. Annexin V might be labeled with a fluorochrome to enable visualization of cells at the early stage of apoptosis. After the MCF-7 cells were treated with Linola straw extracts, phosphatidylserine was localized on the outer leaflet of plasma membrane (**Figure 6**). The morphology of nuclei (stained with Hoechst) of the cells with phosphatidylserine present in the outer leaflet of the membrane was altered and chromatin condensation was observed (**Figure 6**).

**FIGURE 7:**
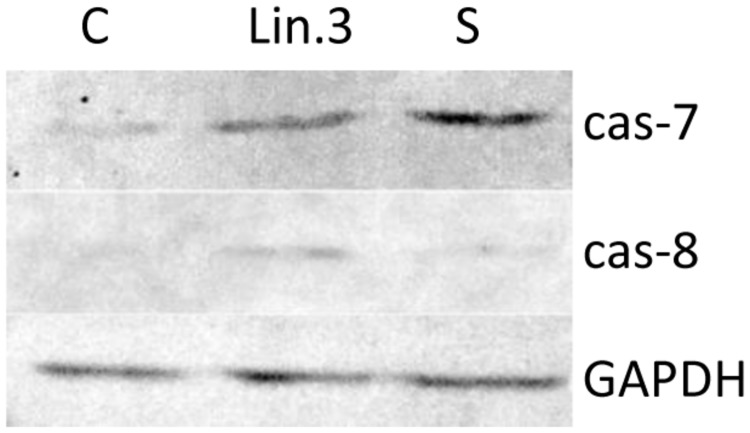
**The expression of caspase-7 and -8 in MCF-7 cells upon Linola flax straw treatment identified in cell lysates and followed by SDS/PAGE and western blot analysis (50 μg of protein per lane).** GAPDH was used as an internal control. Lanes C: control non-treated cells, Lin.3: Linola flax stem extract treated cells, S: staurosporine treated cells.

Apoptosis is a complex activity that mobilizes a number of molecules and is classified into caspase-dependent or caspase-independent mechanisms. To examine the molecular mechanism underlying apoptosis process, activation of caspase-7 and caspase-8 in response to flax straw extract was also analyzed by western blotting assay. The application of mouse monoclonal antibody targeted to active caspase- 7 and rabbit polyclonal antibody targeted to active caspase-8 revealed positively stained MCF-7 cells after 6 h of exposure to Linola flax straw extract (**Figure 7**). An increase of caspase-7 and caspase-8 activity was noted in human breast carcinoma cells when treated with Linola flax straw extract. Our results were parallelly compared with those observed in the presence of 1 μM staurosporine, a known apoptosis induction agent.

## Discussion

Flax is a crop that is used in many branches of industry and is mainly grown for oil production and fiber processing. Flax metabolites are an object of investigation of many studies, but straw remains less investigated. Straw of flax varieties which are grown for oil production are leftovers that contribute to the waste material. Interestingly, they are a source of many health-oriented metabolites of the phenylpropanoids pathway. We examined straw of three transgenic flax types (W92, GT and L) and a non-transgenic one (Linola), and our analysis revealed the presence of *C*-glucoside derivatives of flavonoids, including vitexin, orientin, and isoorientin. Isoorientin was the most abundant metabolite in the straw extracts. Some differences were observed in the level of identified flavonoid *C*-glucosides in all flax types in comparison to the control, but GT, W92, and L flax modifications did not significantly influence the content of flavonoid *C*-glucosides in straw. Further biochemical analysis of flax straw of different flax types revealed inter alia the presence of catechins, ferulic acid, gallic acid, caffeic acid, chlorogenic acids, *p*-coumaric acid, vanillic acid, vanillin, 4-hydroxybenzaldehyde, syringaldehyde, and apigenin derivatives (data not published). Additionally, the levels of the identified compounds were altered depending on the different stages of stem development. Mierziak et al. (2014) studied the biochemical composition of flax stems of W92, GT, and W92 × GT crossbreed plants, and observed an altered level of flavonoid *C*-glucosides in comparison to the control. Similarly, isoorientin was the most abundant compound in flax stems, but our analysis revealed higher amounts of this flavonoid in flax straw (9.01 mg/gFW in comparison to 1 mg/gFW for Linola flax). Interestingly, the level of vitexin was around 10 times lower in our studies. These analyses show the different biochemical composition of flax stems and straw of the same flax types.

For the purpose of these studies, we focused on the identification and determination of the level of flavonoid *C*-glucosides, as it was already reported that they possess inhibitory activity toward human cancer cells in *in vitro* studies. Vitexin has been shown to exhibit apoptotic actions on human U937 human leukemia cells (Lee et al., 2012). Another study showed that orientin and vitexin from *Trollius chinensis* possessed antitumor effects that may be associated with the regulation of the apoptosis-related gene expression of p53 and bcl-2 in esophageal cancer (An et al., 2015). The leaf extract of *Celtis australis* L. *and Celtis occidentalis* L. exhibited cytotoxic activity against human hepatocellular carcinoma, colon adenocarcinoma and gastric carcinoma cell lines (El-Alfy et al., 2011).

The use of flax straw, rich in flavonoid *C*-glucosides, for inhibiting the growth of human breast cancer cells is therefore reasonable. Our analysis indicated that flax straw extracts of different flax types are able to inhibit the proliferation of MCF-7 cells (up to 30%) and are cytotoxic toward this cell line (up to 70% decrease in protein content). The inhibition rate was proportionate to the dose and was more significant at 48 h than at 24 h of treatment. To detect synergism, we used only pure compounds in order to control the working concentrations and avoid effects due to unknown components, as it happens when whole extracts are used. Similarly, treatment of MCF-7 cells with flavonoid *C*-glucoside pure compounds reduced the proliferation and was cytotoxic toward human breast carcinoma cells. The inhibition rates of all compounds tested, vitexin, orientin and isovitexin, were comparable in the MTT proliferation test (up to 60% reduction). Isovitexin was the most effective in the cytotoxic assay (up to 89% reduction). We observed moderate cytotoxicity in MCF-7 cells after treatment with flax straw extract in comparison with the individual flavonoid *C*-glucoside treatment, supporting the hypothesis that there is no synergistic effect of action. Nevertheless, we assume that *C*-glucosides of flavonoids mainly contribute to the inhibitory activity toward MCF-7 cells, as the activity of these metabolites translates into the activity of the extract. Interestingly, we observed that the smaller volumes of some straw extract samples caused a pro- proliferative effect. A similar phenomenon was observed after treatment of breast cancer cell line BT-20 and bone cancer cell line MG63 with relatively low concentrations of root bark extract of *Persea americana* and leaves extract of *Hunteria umbellata*, traditional Nigerian medicinal plants, which caused an increase in proliferation up to 8 and 27%, respectively (Engel et al., 2011).

To elucidate the effect of flax straw extracts on the induction of apoptosis in MCF-7, we performed expression level analysis of crucial genes involved in this process. It is well-known that mitochondria are implicated as they are one of the apoptotic targets, and the balance between the expression levels of Bcl-2 and Bax proteins determines whether the cell responds to an apoptotic signal. Bax, a pro-apoptotic factor, integrates into the outer mitochondrial membrane and leads to the release of cytochrome-c from mitochondria, which further activates caspases-9 (Thomas et al., 2000). In contrast, Bcl-2 prevents this process by preserving mitochondrial integrity and promotes cell survival (Jeong and Seol, 2008). To reveal the effect of flax straw extracts on expression of Bcl-2 and Bax, RT-PCR was performed. It was observed that flax straw extracts were involved in the upregulation of Bax and downregulation of Bcl-2. There are two core signaling pathways that are involved in apoptosis, the intrinsic and the extrinsic pathway. The intrinsic pathway is a signal transduction pathway involving the mitochondria and the Bcl-2 family, which was observed in the MCF-7 cells treated with flax straw extracts, and therefore we suggest that flax straw extract might trigger the intrinsic pathway of apoptosis.

Caspase activation is considered to be a key hallmark of apoptosis. Caspases, a conserved family of cysteine proteases, are the central components of the apoptotic response that irreversibly commit a cell to die. Breast carcinoma cells of the MCF-7 line have lost expression of caspase 3 as a result of a 47 base pair deletion within exon 3 of the casp-3 gene (Liang et al., 2001). Despite the absence of detectable caspase 3, MCF-7 cells have been shown to undergo apoptosis. Moreover, it is known that caspase 7 is highly related to caspase 3 and shows the same substrate specificity *in vitro* (Talanian et al., 1997). We assessed the effect of flax straw extracts on the cascade of caspases, and the mRNA expression levels of caspase 7, 8, and 9 were determined in our experiment. Results from the present study demonstrated that mRNA expression levels of caspase 7, 8, and 9 were increased. In addition, the present study suggests that the extrinsic pathway may in part have contributed to the flax straw extract-induced apoptosis, as demonstrated by an increased expression level of caspase-8 in MCF-7 cells. It is suggested that caspase-8 might activate Bid protein (BH3 interacting domain), which might be a molecular bridge to intrinsic pathway of apoptosis, as it binds to the activated Bax protein on mitochondria surface, and thus contribute to the pore generation in this organelle (Kantari and Walczak, 2011). Interestingly, increased expression of p53 was not detected in the MCF-7 cells used in the present experiments, and the mdm2 expression was elevated in the late apoptosis. p53 is one of the most powerful tumor suppressor genes in human cancers, and both extrinsic and intrinsic apoptotic pathways are activated by p53, while mdm2 is an important negative regulator of p53 and is the primary cellular inhibitor of p53 (Maximov and Maximov, 2008). Our findings suggest that flax straw extracts induce p53-independent apoptosis through downregulation of Bcl-2. Similarly, it was shown that curcumin caused apoptosis in melanoma cell lines and in a human colon cancer cell line in a p53-independent pathway (Bush et al., 2001; Watson et al., 2010). We did not observed the significant changes in the transcript level of annexin V gene.

For further confirmation of apoptosis, we performed the method that uses the binding of FITC-labeled annexin V to phosphatidylserine which is exposed on the surface of apoptotic cells but not on viable cells. Our experiments indicated that flax straw extract induces apoptotic processes in MF-7 cells, as these cells were annexin V-positive. Moreover, the nuclei morphology visualized with Hoechst staining also indicates an apoptotic process upon flax straw treatment. Part of the flax straw treated cells exhibited a homogeneous intense staining of the nucleus or nuclear fragments, that is characteristic for apoptotic cells with nuclear condensation. All cells with nuclear condensation were also stained with annexin V at the cell membrane, whereas, none of the cells with a euchromatic nucleus were annexin V-positive. Therefore, we suggest that annexin V-positive cells are undergoing apoptosis.

Additionally, the apoptotic process occurrence upon flax straw extract treatment was confirmed by the assessment of the protein level of caspase-7 and caspase-8. The flax straw extract treatment resulted in an increase of activated form of the proteins of both examined caspases. We suggest that this treatment results in the apoptosis process activation, as the staurosporine, known a known apoptosis induction agent, treated MCF-7 cells exhibited the same level of caspase-7 and -8 protein as the flax straw extract- treated cells (**Figure 7**). Flax straw extract induce apoptosis via caspase- dependent mechanism.

Taken together, the results of the present study suggest that flax straw extracts inhibit the proliferation of MCF-7 cancer cells by increasing apoptosis and might provide a useful alternative to cancer chemotherapy. Due to the adverse effects and resistance of many anticancer agents that have been developed, there is a great interest in the use of plant-based compounds to develop safe and efficient antitumor factors.

## Author Contributions

AK, JS made substantial contributions to conception and design of the study and to data interpretation; AK performed UPLC analysis, MC carried out plant extracts preparation, cell culture proliferation, cytotoxicity experiments, gene expression levels analysis, Annexin V staining, western blot analysis and made substantial contributions to acquisition and data analysis and wrote the manuscript. JM performed the real-time PCR analysis, western blot analysis and preformed statistical analysis. All authors reviewed and approved the manuscript.

## Conflict of Interest Statement

The authors declare that the research was conducted in the absence of any commercial or financial relationships that could be construed as a potential conflict of interest. The reviewer IB and handling Editor declared their shared affiliation, and the handling Editor states that the process nevertheless met the standards of a fair and objective review.
