# Supplementary material for: Flavonoid C-glucosides Derived from Flax Straw Extracts Reduce Human Breast Cancer Cell Growth In vitro and Induce Apoptosis
Source: Front Pharmacol. 2016 Aug 31;7:282. doi: 10.3389/fphar.2016.00282 (PMC5006111; doi:10.3389/fphar.2016.00282)
Supplement: Supplementary file 1 [file Table_1.DOCX]

**TABLE 1** Primer sequences for real-time RT-PCR reactions.

| Gene | Forward primer | Reverse primer |
| --- | --- | --- |
| bcl2 | 5’-TGGCCTTCTTTGAGTTCG-3’ | 5’GTACAGTTCCACAAAGGCAT-3’ |
| bax | 5’-CGAACTGGACAGTAACATGG-3’ | 5’-CAGTTTGCTGGCAAAGTAGA-3’ |
| caspase-7 | 5’CATGCGATCCATCAAGACCA-3’ | 5’-GGAAGCACTTGAAGAGCG-3’ |
| caspase-8 | 5’‑AATTAATAGACTGGATTTGCTGATTAC-3’ | 5’-CCTCAATTCTGATCTGCTCAC-3’ |
| caspase-9 | 5’-TTGTCGAAGCCAACCCTA-3’ | 5’-GCCAAATCTGCATTTCCC-3’ |
| p53 | 5’-ACATGACGGAGGTTGTGA-3’ | 5’-CACCACCACACTATGTCG-3 |
| mdm2 | 5’-AAGGAGAGCAATTAGTGAGAC-3’ | 5’‑TGCTACTGCTTCTTTCACAAC-3’ |
| annexin V | 5’-CTTTATTTCAGGCTGGAGAACTTA-3’ | 5’-ATCCTGATATAGTCATGTACTTGT-3’ |
| GAPDH | 5’‑AGGTCGGAGTCAACGGAT-3’ | 5’-TCCGGAAGATGGTGATG3’ |
